# Identifying Age Based Maturation in the ERP Response to Faces in Children With Autism: Implications for Developing Biomarkers for Use in Clinical Trials

**DOI:** 10.3389/fpsyt.2022.841236

**Published:** 2022-05-09

**Authors:** Sara Jane Webb, Iris Emerman, Catherine Sugar, Damla Senturk, Adam J. Naples, Susan Faja, Jessica Benton, Heather Borland, Carter Carlos, April R. Levin, Takumi McAllister, Megha Santhosh, Raphael A. Bernier, Katarzyna Chawarska, Geraldine Dawson, James Dziura, Shafali Jeste, Natalia Kleinhans, Michael Murias, Maura Sabatos-DeVito, Frederick Shic, James C. McPartland, the Autism Biomarkers Consortium for Clinical Trials

**Affiliations:** ^1^Center on Child Health, Behavior, & Development, Seattle Children's Research Institute, Seattle, WA, United States; ^2^Department of Psychiatry & Behavioral Sciences, University of Washington School of Medicine, Seattle, WA, United States; ^3^Department of Biostatistics, University of California, Los Angeles, Los Angeles, CA, United States; ^4^Department of Psychiatry & Biobehavioral Sciences, University of California, Los Angeles, Los Angeles, CA, United States; ^5^Department of Statistics, University of California, Los Angeles, Los Angeles, CA, United States; ^6^Yale Child Study Center, Yale University, New Haven, CT, United States; ^7^Harvard Medical School, Harvard University, Boston, MA, United States; ^8^Department of Neurology, Boston Children's Hospital, Boston, MA, United States; ^9^Duke Center for Autism and Brain Development, Duke University, Durham, NC, United States; ^10^Department of Psychiatry & Behavioral Sciences, Duke University, Durham, NC, United States; ^11^Yale Center for Clinical Investigation, Yale University, New Haven, CT, United States; ^12^Department of Neurology, Children's Hospital of Los Angeles, Los Angeles, CA, United States; ^13^Center on Human Development and Disabilities, University of Washington, Seattle, WA, United States; ^14^Department of Radiology, University of Washington School of Medicine, Seattle, WA, United States; ^15^Medical Social Sciences, Northwestern University, Chicago, IL, United States; ^16^Department of Pediatrics, University of Washington School of Medicine, Seattle, WA, United States

**Keywords:** autism spectrum disorders, biomarkers, clinical trial methods, ERP, face processing, P100, N170, age

## Abstract

Recent proposals have suggested the potential for neural biomarkers to improve clinical trial processes in neurodevelopmental conditions; however, few efforts have identified whether chronological age-based adjustments will be necessary (as used in standardized behavioral assessments). Event-related potentials (ERPs) demonstrate early differences in the processing of faces vs. objects in the visual processing system by 4 years of age and age-based improvement (decreases in latency) through adolescence. Additionally, face processing has been proposed to be related to social skills as well as autistic social-communication traits. While previous reports suggest delayed latency in individuals with autism spectrum disorder (ASD), extensive individual and age based heterogeneity exists. In this report, we utilize a sample of 252 children with ASD and 118 children with typical development (TD), to assess the N170 and P100 ERP component latencies (N170L and P100L, respectively), to upright faces, the face specificity effect (difference between face and object processing), and the inversion effect (difference between face upright and inverted processing) in relation to age. First, linear mixed models (LMMs) were fitted with fixed effect of age at testing and random effect of participant, using all available data points to characterize general age-based development in the TD and ASD groups. Second, LMM models using only the TD group were used to calculate age-based residuals in both groups. The purpose of residualization was to assess how much variation in ASD participants could be accounted for by chronological age-related changes. Our data demonstrate that the N170L and P100L responses to upright faces appeared to follow a roughly linear relationship with age. In the ASD group, the distribution of the age-adjusted residual values suggest that ASD participants were more likely to demonstrate slower latencies than would be expected for a TD child of the same age, similar to what has been identified using unadjusted values. Lastly, using age-adjusted values for stratification, we found that children who demonstrated slowed age-adjusted N170L had lower verbal and non-verbal IQ and worse face memory. These data suggest that age must be considered in assessing the N170L and P100L response to upright faces as well, and these adjusted values may be used to stratify children within the autism spectrum.

## Introduction

Face processing is a foundational skill that supports social communication and has been proposed as a promising biomarker related to social function. Significant attention has been paid to the face processing skills of individuals with ASD, with group effects suggesting altered visual attention to facial features [e.g., (1)] and worse memory for faces [e.g., (2)]. While face processing delays have been proposed as a core feature in early ASD with negative developmental consequence on later social functioning (3, 4), significant heterogeneity exits including individual performance overlap with non-ASD groups [e.g., (5, 6); for discussion: (7)].

### ERPs to Faces

The neural sources of face processing have been well-explored [e.g., (8–10)], with event-related potential (ERP) demonstrating early temporal differences in the processing of faces in the visual processing system. Bentin et al. (11) first identified the N170 component to faces as a negative-going voltage deflection recorded over the occipitotemporal scalp in adults; the N170 was greater in amplitude and faster in latency to faces than to other stimuli categories, and larger in amplitude but slower in latency to inverted compared to upright faces [A general review of the properties of the N170 in response to manipulation of the face is available *via*: (12)].

Children also display a developmental version of the N170, a negative ERP component that is similarly largest in amplitude at posterior temporal electrodes and larger in amplitude for eyes and upright faces in comparison to other non-face stimuli [e.g., (13–16)]. The N170 becomes markedly faster from 4 to 9 years with a less steep change between 10 and 15 years of age [e.g., Figure 7, (16)]. Mares et al. (17) suggest a lack of the adult-N170 inversion effect in childhood (6–11 years) (17). However, the N170 inversion effect [longer latency for inverted faces thought to index holistic processes (18)] was found in a sample of 8 to 9-year-old children when acquired during an explicit face recognition task but not until after 11 years in an implicit task. Thus, the inversion effect likely emerges during middle childhood and is vulnerable to tasks constraints. A further confound in developmental analyses is the presence of a *bifid* N170 peak present in 65% of children <12 years [Figure 6, (16)] or in children <9 years (14). Variability in waveform morphology and peak “peaking” protocols likely creates methodological inconsistencies that may further impact general interpretation of developmental trends.

In addition, the P100 is a positive deflection that is thought to index attentional processes, but also shows face sensitivity with more positive amplitude and shorter latencies to faces than other stimuli [e.g., (19, 20)], and faster latencies to upright compared to inverted faces (19, 21). In children, the inversion effect may be more prominent at the P100, with smaller amplitude but faster processing of upright compared to inverted faces (16). Less is known about the age-related changes in the P100 to faces despite it often being used to anchor the N170 window [e.g., (14)].

### ERPs in Individuals With ASD

Both the N170 and the P100 have been found to differ between groups of individuals with ASD compared to typical development. Longer latencies for N170 responses to upright faces (suggesting slower processing) relative to neurotypical peers have been observed in ASD (22, 23). This slower N170 upright face latency effect has been found with ASD children aged 6–11 years in the ABC-CT sample (24) as well as 9–17-year-old children (25). Group differences have also been found in the P100 with slower latency to upright faces in children with ASD compared to children with TD [6–11 year old children: (24); 5–30 years: (26)].

A reduced inversion effect has been found in adults with ASD for P100 and N170 amplitude but not for the latency of either components (27). Similarly main effects of inversion but not group differences were found in samples that include children and adults [P100 latency (26)], and children aged 8–13 years [P100 latency: (28)]. In contrast, no inversion effects were found on N170 latency in three other developmental reports (25, 26, 28).

The N170 latency variability also appears to associate with social-emotional function in areas such as social competence, distress, empathy, emotional sensitivity, anxiety, introversion, shyness, and social withdrawal [for review: (23)] as well as face memory (24). Thus, while it is not clear that a reduced or slowed neural response is characteristic of individuals who reflect the broad autism spectrum, variability in this response may be related to aspects of the clinical phenotype of autism and other subdomains of social ability.

### Developmental ERPs to Faces

While general age-based decreases in latency of ERP components have been reported, less is known about age-based trajectories in autistic individuals. Kang et al. note the significance of age as a moderator variable in their meta-analysis, with larger differences between ASD and TD in older youth and/or adult samples (23). In empirical reports, Neuhaus et al. found both the P100 and the N170 latency were significantly influenced by age, with the P100 showing an interaction between stimulus, orientation and age, and the N170 showing a main effect of age (26). Similarly, Hileman reported age effects for the P100 but not the N170, suggesting improved processing efficiency with age in attentional systems (25). In our ABC-CT sample, we found a significant relationship between age and the P100 (TD *r* = −0.215; ASD *r* = −0.185, *p*s < 0.01) and the N170 latency (TD *r* = −0.369; ASD *r* = −0.350, *p*s < 0.01), with faster latencies in older children (24). While it is clear there is age-based change in the ERP components related to face processing, precise metrics of growth, especially in ASD, have not been articulated.

### Aims

If ERP markers are to be used as biomarkers in developmental populations, detailed analysis of chronological age-based maturation are necessary to evaluate whether or not stratification thresholds will required age-based adjustment (as used in many standardized assessments). The primary aim of this analysis is to characterize the age-based changes in neural systems represented by the N170 and the P100 *latency* response to face processing in the ABC-CT sample of 280 children with ASD compared to 119 children with TD. We focus on latency given that peak amplitude is impacted by trial-to-trial latency variability and previous work suggests a reliable decrease in peak processing time in childhood. As a secondary aim, we investigate the age-based characterization of N170 latency face specificity effect (FSE), which provides a metric of the neural specialization to faces by calculating the difference between the processing of upright faces compared to upright objects. We also include the inversion effect (IE) (upright face compared to inverted face). Third, we use age-adjusted N170 and P100 responses to identify whether these biomarkers can be used for stratification.

## Methods

### Protocol

Details about the ABC-CT (Autism Biomarkers Consortium for Clinical Trials) protocol are published (29, 30). Data are available via (NDA study #2288) and the project is listed in ClinicalTrials.Gov (NCT02996669).

Briefly, the first phase of the ABC-CT included children aged 6.0–11.5 years, with 280 children with ASD (meeting gold standard ASD diagnostic criteria and full-scale IQ between 60 and 150) and 119 typically developing children (confirmed to be free of elevated psychiatric, psychological, or developmental concerns and full-scale IQ between 80 and 150) assessed at 5 sites using clinician, caregiver, and lab-based measures of social functioning and a battery of EEG and eye-tracking (ET) tasks. The protocol was administered during 2-day visits, with the EEG on the second day. Participants provided data at three timepoints (T1, T2 +6 weeks, and T3 +6 months). Distance in days between timepoint is provided in the Section Protocol, (Supplementary Table 1). Using a central IRB, informed consent/assent was obtained from guardians/participants after all procedures had been fully explained and the opportunity to ask questions was offered.

### Participant Characteristics

Table 1 provides demographic characteristics of the enrolled ABC-CT sample at Time 1 (“All”), for the subsample contributing valid data to the analysis (“Faces”), and for the subsample not contributing valid data to the analysis (“No data”). Clinical observation was provided through use of the Differential Abilities Scale (DAS: Full Scale IQ, Verbal IQ, Non-Verbal IQ), Autism Diagnostic Observation Schedule-2 [Comparison Score (CS)], and the NEPSY Face Memory task. Parents provided additional characterization through the Vineland Adaptive Behavior Scales-3 (VAB3) interview (Socialization Standard Score, Communication Standard Score), and parent report questionnaires including the Social Responsiveness Scale [SRS Social Communication and Interaction T-Score (SCI) and Restrictive Interest and Repetitive Behavior T-Score (RIRB)], and Pervasive Developmental Disorder and Behavior Inventory [PDDBI Social Approach T-Score (SocApp)]. Further participant characterization is available in Faja et al. (31). In this report, age was based on the day of the EEG visit. Age did not differ between the ASD and TD groups when considering the total sample at Time 1 (*F* = 0.045, *p* = 0.833), nor when including only those with ERP data for the Faces assay (*F* = 1.623, *p* = 0.20).

**Table 1:** Summary of participant characteristics at Time 1.

**Time 1**	**ASD All**	**TD All**	**ASD Faces**	**TD Faces**	**ASD No data**	**TD No data**
*N* total	280	119	252	118	28	1
*N* female	65	36	60	36	5	0
% female	23%	30%	24%	31%	18%	0%
Age in yrs	8.6 (1.6)	8.5 (1.6)	8.7 (1.6)	8.5 (1.6)	7.4 (1.4)	NA
DAS Verbal IQ	96.0 (20.7)	116.3 (11.2)	97.7 (19.8)	116.4 (11.2)	80.5 (22.1)	99 (NA)
DAS NonV IQ	97.5 (16.9)	112.2 (14.1)	98.7 (16.7)	112.4 (13.8)	87.3 (15.5)	81 (NA)
DAS Full IQ	96.6 (18.1)	115.1 (12.6)	98.1 (17.7)	115.4 (12.3)	83.0 (16.4)	86 (NA)
ADOS CSS	7.6 (1.8)	1.6 (0.9)	7.6 (1.8)	1.6 (0.9)	8.0 (1.3)	1 (NA)
VABS3 Soc SS	69.9 (16.1)	104.6 (9.2)	70.6 (15.9)	104.6 (9.2)	63.5 (17.5)	NA
VABS3 Com SS	76.4 (15.1)	103.4 (9.2)	77.3 (14.7)	103.4 (9.2)	68.6 (16.5)	NA
SRS-2 SCI T	72.7 (10.8)	42.5 (5.1)	72.4 (11.0)	42.5 (5.1)	75.2 (8.2)	38 (NA)
SRS-2 RIRB T	73.7 (12.2)	44.0 (3.7)	73.4 (12.4)	44.0 (3.7)	76.4 (9.4)	43 (NA)
PDDBI SocApp T	54.2 (9.3)	69.8 (3.0)	54.4 (9.4)	69.9 (3.0)	52.4 (8.4)	65 (NA)
NEPSY face memory SS	7.9 (3.7)	10.5 (3.5)	8.1 (3.7)	10.6 (3.5)	5.9 (3.0)	8 (NA)

*Mean and standard deviation are presented for assessments for the full sample (All), the subset of participants providing at least one valid data point contributing to the analyses (Faces), and the subset of participants providing no valid data points (No data).*

*DAS, Differential Ability Scale; NonV, Non Verbal; ADOS, Autism Diagnostic Observation Schedule; CSS, Calibration Severity Score; VAB3, Vineland Adaptive Behavior Scales-3; Soc, Socialization; Com, Communication; SS, Standard Score; SRS, Social Responsiveness Scale; RIRB, Restricted Interests and Repetitive Behavior subdomain; SCI, Social Communication and Interaction subdomain; T, T-Score; PDDBI, Pervasive Developmental Disorder Behavioral Inventory; SocApp, Social Approach Behaviors Domain*.

### EEG Acquisition

All procedures for standardization are available via request and in Webb et al. (30). All sites had an EGI 128 channel acquisition system, with either Net Amps 300 (*n* = 3) or 400 (*n* = 2), 128 electrode EGI HydroCel Geodesic Sensor Nets, Logitech Z320 Speakers, Cedrus StimTracker (for visual presentation timing), and a Dell 23″ monitor. A standard acquisition setup was implemented: 1,000 Hz sampling rate, 0.1–200 Hz filter, EGI MFF file format, onset recording of amplifier and impedance calibrations, and a 0.1 Hz digital high pass filter post-acquisition. EPrime 2.0 was used for experimental control.

The ERP Faces Assay included 6 blocks of 36 stimuli, comprised of 3 neutral female faces presented upright (FaceUp) or inverted (FaceInv) (32) and 3 houses presented upright (HouseUp) with visual angle was 12.3 × 9.3 degrees. The experiment included 216 trials, acquired in 6 blocks of 36, resulting in 72 trials per condition. Each trial consisted of a fixation crosshair (500–650 ms), stimulus (500 ms), and blank screen (500–650 ms). A schematic of the assay is presented in Supplementary Figure 1. During acquisition, the experimenter coded the participant's behavior for attention and compliance and any trial in which the participant did not attend to the image was discarded.

### EEG Processing

Post-acquisition, EEG data was processed using the PREP algorithm (33) to remove line-noise, re-reference to a robust average reference, and interpolate bad channels relative to this reference. We then bandpass filtered the EEG at 0.1–30 Hz. Trials were segmented to 200 ms before and 500 ms after stimulus onset and unattended trials were removed. Baseline correction was applied using the 200 ms pre-stimulus interval. Artifact detection was done using the ERPLab function *pop_artextval* (34) with a threshold of 150 μV and a time window of −200 to 500 ms.

A participant's data was included if they had ≥21 artifact free and attended FaceUp trials. We focused on the right posterior-temporal region (RPT) for both components, which was created by averaging 5 channels (89, 90, 91, 95, 96); the analysis of lateral leads for the P100 (60–200 ms) and N170 (120–400 ms) and is consistent with prior publications [e.g., (16, 25, 26, 35, 36). The net layout is presented in Supplementary Figure 2. Trials were averaged by stimulus condition (FaceUp, FaceInv, HouseUp). The P100 Latency (P100L, 60–200 ms) and N170 Latency (N170L, 120–400 ms) peak amplitude and latency were identified using an automated algorithm and then visually inspected and adjusted at the individual level for the region of interest *via* manualized definitions available in Section EEG Processing. Supplementary Figure 3 depicts the grand average waveforms. Supplementary Tables 2, 3 provides information on missing data by group, timepoint, and variable of interest. Average number of trials (artifact free, attended) are presented in Supplementary Table 4.

### Analytic Plan

For Aim 1, primary dependent variables were the N170L and P100L responses to FaceUp. For Aim 2, we also examined: (1) the face specificity effect for both components (FSE), which is the difference in the peak latency value for upright faces minus upright houses and (2) the inversion effect (IE), which is the difference in peak latency for upright faces minus inverted faces.

#### Age Based Development

Linear mixed models (LMM) were constructed for each relevant ERP component using all available data. To determine the variance structure in the ASD and TD groups, separate models were constructed in each group before the combined model was constructed. Final models had the fixed effects of timepoint, group, and mean-centered age at acquisition, and participant level random intercepts with different random effect variance structures for TD and ASD. Confidence intervals for fixed effects were estimated using likelihood profiles. Timepoint was included as a factor in the model to adjust for potential exposure effects of repeat testing. Random slopes were not included in the model because each participant only had, at most, three timepoints, two of which (T1 and T2) were close in time.

More complex models were also considered in the characterization of age-based changes in N170L and P100L in response to FaceUp. Models were compared using Akaike Information Criterion (AIC) values, with lower AIC values suggesting better model fit. For both FaceUp N170L and P100L, the models described above had a lower AIC than models with the same random and fixed effects but with the addition of a fixed interaction effect between group and age at testing. Adding a quadratic term for age at testing to the models described above also resulted in models with higher AIC values for both models. AIC values for the three types of models that were assessed are available in Supplementary Table 5.

#### Age Adjustment

The purpose of residualization using the TD group is to understand age-based distributional characteristics of the ASD sample independent of developmental or maturational expectations. That is, the residualization is derived based on the developmental trajectory in the TD group. Hence it can be used to investigate whether the latencies in the ASD sample are slower or faster than what they would be expected to be based on age. Even though they are derived based on age-specific comparisons, the residulization values are age-free.

**Figure 1:**
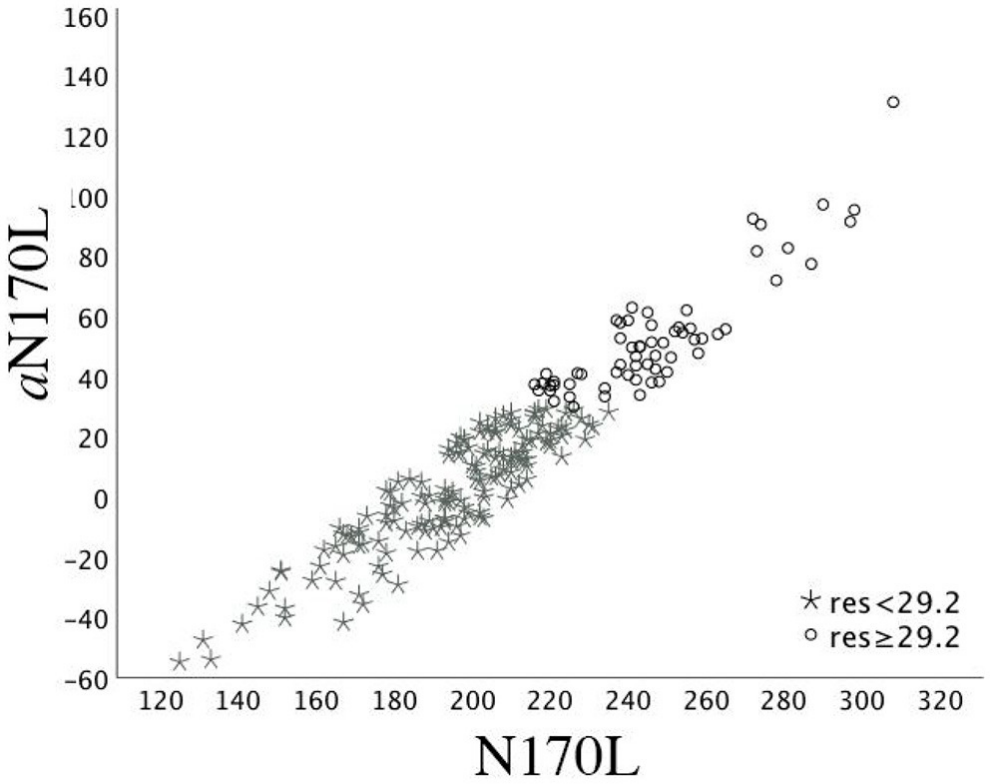
Time 1 N170L and *a*N170L for the ASD group. Relation between the raw Time 1 N170L (x-axis) and the age-adjusted N170L (aN170L)(y-axis) in response to upright faces. Children with ASD and slowed age-adjusted N170s are identified by black open circles; Children with ASD and standard age-adjusted N170s are identified by gray stars. The use of the residual cutpoint of >29.2 reflects the ASD sample overlap with 10% of the TD group.

To create these values, random intercept models with fixed effect of age at testing and random effect of participant were fitted using all available data points in the TD group and then used to calculate age-based residuals in the TD and ASD groups. A positive residual value in the ASD sample indicates that the latency was slower than would be expected in a typically developing child of the same age. A negative residual value indicates that the latency was faster than would be expected in a typically developing child of the same age. See Figure 1 for the relation between the (raw) N170L FaceUp values and the age-adjusted or residualized N170L (aN170L) FaceUp values. Raw values for the N170L and P100L for the FaceUp are presented in Table 2. Raw values for FSE and IE are presented in Supplementary Tables 6, 10; ICC values are presented in Supplementary Table 7 and adjusted values are presented in Supplementary Table 11.

**Table 2:** Summary statistics for raw and residualized N170L and P100L in response to upright faces.

	**FaceUp N170L**		**FaceUp** ***a*****N170L**		**FaceUp P100L**		**FaceUp** ***a*****P100L**	
	**TD**	**ASD**	**TD**	**ASD**	**TD**	**ASD**	**TD**	**ASD**
*N*	336	624	336	624	336	623	336	623
Missing	21	216	21	96	21	217	21	97
Mean	193.60	206.23	0.19	14.01	117.57	121.74	0.17	4.76
Median	193.0	203.5	−1.82	11.45	117.0	119.0	−0.38	1.95
SD	27.13	34.16	25.04	32.31	13.12	16.89	12.60	16.76
Skewness	0.33	0.92	0.47	1.14	0.96	1.20	0.86	1.23
SE of Skewness	0.13	0.010	0.13	0.10	0.13	0.10	0.13	0.10
Kurtosis	0.26	2.97	0.93	3.54	4.35	2.07	4.51	2.16
SE of Kurtosis	0.27	0.20	0.27	0.20	0.27	0.20	0.27	0.20
Min	125.0	125.0	−68.0	−60.0	82.0	82.0	−41.0	−32.0
Max	276.0	393.0	88.0	185.0	189.0	192.0	69.0	72.0
% 10	158.7	166.5	−28.61	−23.21	103.7	104.0	−13.27	−12.61
% 25	177.0	186.0	−15.51	−7.15	110.0	111.0	−6.85	−5.24
% 30	181.0	190.0	−13.82	−2.60	112.0	113.0	−5.53	−3.83
% 50	193.0	203.5	−1.82	11.45	117.0	119.0	−0.38	1.95
% 70	204.9	219.0	12.75	26.01	122.0	126.0	5.18	8.77
% 75	208.0	224.0	16.90	30.97	124.0	128.0	6.77	10.41
% 90	229.30	246.0	29.20	50.68	130.30	145.0	12.57	27.28

*The model used to calculate residuals was a mixed effect model fitted in the TD group. Age was a fixed effect and participant ID was a random effect. Skewness is close to 0 when the distribution is symmetrical, negative when the left tail of the distribution is longer, and positive when the right tail of the distribution is longer. Larger values of kurtosis indicate heavier tails (kurtosis = 3 for a univariate normal distribution)*.

#### Stratification

We then investigated the potential use of these age-adjusted ERP values for stratification. That is, if distributions of the age-adjusted residuals contain a tail, this may represent distinct neural subgroups of children with ASD. To this end, a cutoff point for each adjusted-ERP component was calculated based on the upper 10% of all available age-adjusted residual values from the TD group. We identified the participants in the ASD group whose T1 values were greater than or less than that age-adjusted cutoff. We compared clinical characterization for autistic children stratified by the cutpoint using unadjusted ANOVAs.

## Result

### Age Based Development Using Linear Mixed Models for Upright Faces

Based on preliminary exploration of P100L and N170L, it was determined that there were substantial differences in variance structure between the ASD and TD groups. Models were therefore constructed with differing random effect variance structure for ASD and TD groups when data complexity allowed. Table 3 shows fixed and random effects for the model. It should be noted that *p*-values have not been included in descriptions of fitted LMMs; this is a deliberate omission (37).

**Table 3:** Random and fixed effects for N170 latency and P100 latency to upright faces.

**N170 Latency FaceUp**			**P100 Latency FaceUp**		
**960 observations, 370 participants**			**959 observations, 369 participants**		
**Fixed effects**	**Estimate (95% CI)**	***t-*value**	**Fixed effects**	**Estimate (95% CI)**	***t-*value**
Intercept	195.4 (191.0, 199.9)	85.9	Intercept	116.8 (114.6, 119.0)	105
Age	−0.018 (−0.022, −0.013)	−7.8	Age	−0.0056 (−0.0079, −0.0033)	−4.7
ASD group	14.4 (9.0, 19.8)	5.2	ASD group	4.61 (1.9, 7.3)	3.3
T2	−3.4 (−6.3, −0.43)	−2.2	T2	0.78 (−0.67, 2.2)	1.1
T3	−3.3 (−6.3, −0.18)	−2.1	T3	0.95 (−0.57, 2.5)	1.2
**Random effects**	**Variance (SD)**		**Random Effects**	**Variance (SD)**	
TD	396.4 (19.9)		TD	93.8 (9.7)	
ASD	645.6 (25.4)		ASD	187.0 (13.7)	
Residual	350.0 (18.7)		Residual	84.9 (9.2)	

*Mixed effect model fits a random y-intercept with differing variances for typically developing (TD) and autism spectrum disorder (ASD) groups*.

The TD and ASD participant variances, estimate, on average, how much the ERP latency component deviates from their age- and timepoint-based predictions across participants, specific to each diagnostic group. Since age at testing has been mean-centered (with sample mean 8.7 years old), the fixed effect intercept represents an estimate of mean ERP component score for typically developing individuals at baseline.

For FaceUp, the average N170L was an estimated 0.018 ms (95% CI: −0.022, −0.013) faster for each additional day of age (Table 3, left) or 6.57 ms per year. Between-participant variance for N170L FaceUp is greater in the ASD group (*SD*_ASD_ = 25.4) than the TD group (*SD*_TD_ = 19.9).

The average P100 Latency FaceUp was an estimated 0.0056 ms (95% CI: −0.079, −0.0033) faster for each additional day of age (Table 3, right) or 2.05 ms per year, adjusting for group and timepoint. Between-participant variance for P100L FaceUp is greater in the ASD group (*SD*_ASD_ = 13.7) than the TD group (*SD*_TD_ = 9.7).

### Age-Adjusted Residuals for Upright Faces

**Figure 2:**
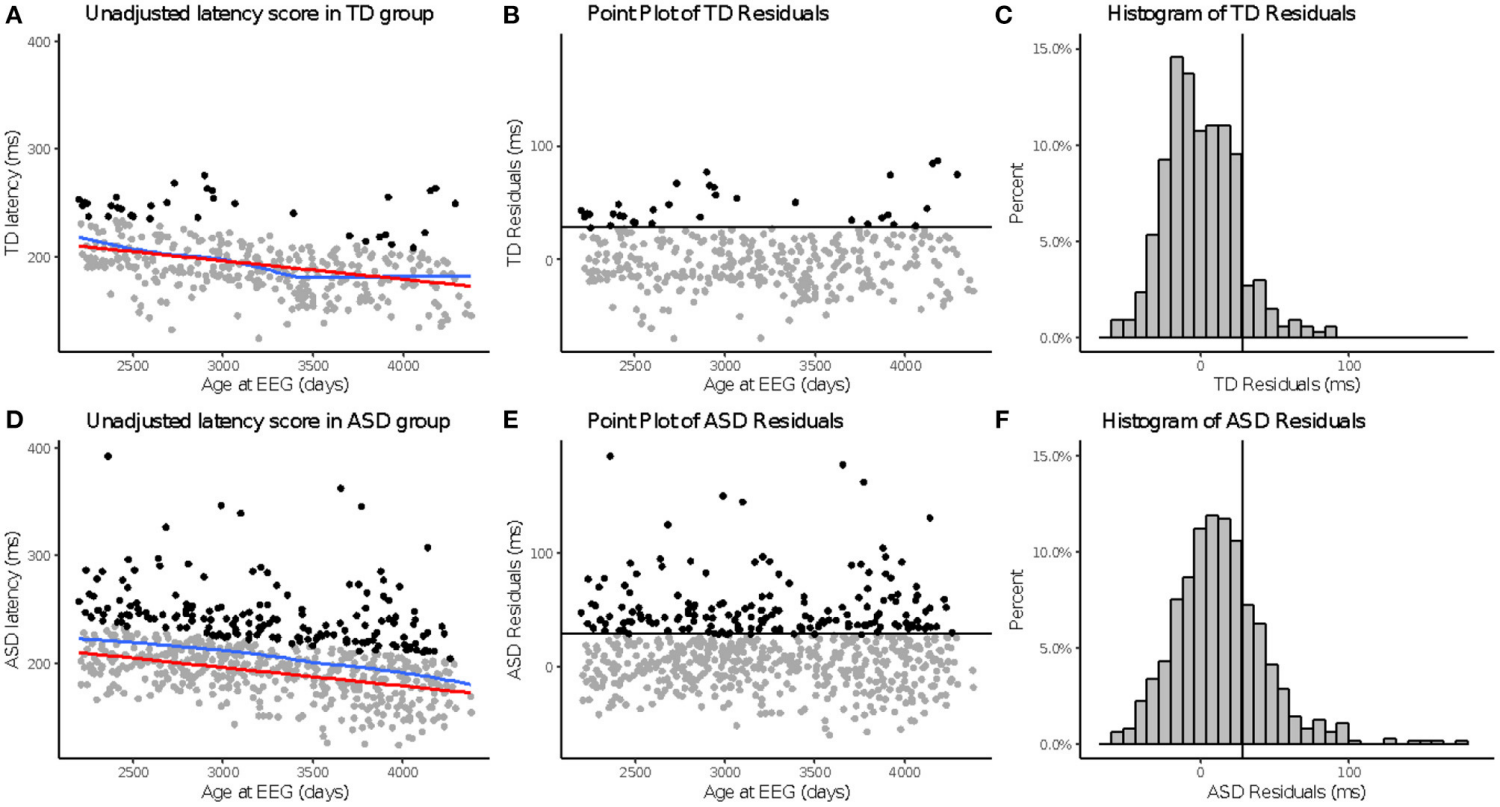
*a*N170 latency to upright faces. Age-adjusted N170L in response to upright faces. Graphs depict the TD group in the top row **(A–C)** and ASD in the bottom row **(D–F)**. **(A,D)** Red line indicates predicted values of N170L based on the fitted model, while the blue line indicates the locally estimated scatterplot smoothing (LOESS) for each group. **(B,E)** Residual values calculated using the fitted model. **(C,F)** Black line indicates a cutoff point derived from the upper 10% of all age-adjusted N170L scores in the TD group. Data points greater than that cutoff point are black **(A,B,D,E)**.

**Figure 3:**
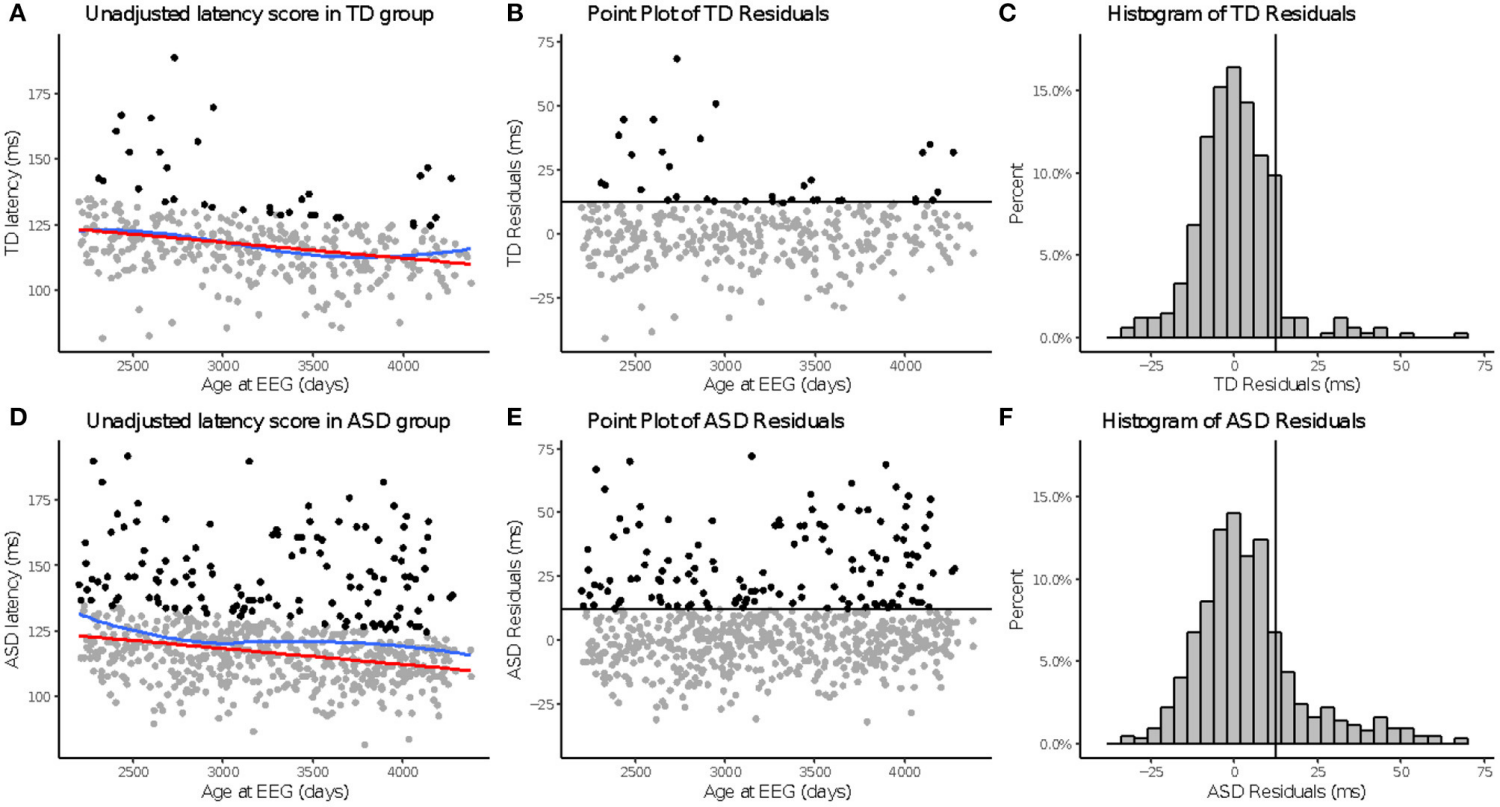
*a*P100 latency to upright faces. Age-adjusted P100L in response to upright faces. Graphs depict the TD group in the top row **(A–C)** and ASD in the bottom row **(D–F)**. **(A,D)**: The red line in column 1 indicates predicted values of P100L based on the fitted model, while the blue line indicates the locally estimated scatterplot smoothing (LOESS) for each group. **(B,E)**: Graphs show the residuals values calculated using the fitted model. **(C,F)**: Black lines indicate a cutoff point derived from the upper 10% of all age-adjusted P100L scores in the TD group. Data points greater than that cutoff point are also in black **(A,B,D,E)**.

Across N170L (Figure 2) and P100L (Figure 3), the distribution of residuals in the ASD group (Figures 2F, 3F) suggest slower than expected N170 latencies in the ASD group (Figure 2C) similar to what we reported for raw values (24).

Overall, there was greater variability in ERP component scores observed between individuals in the ASD group than between individuals in the TD group. Higher between-participant variation is evidence for greater heterogeneity in the FaceUp N170L and P100L components across the ASD participants. This pattern of greater between-participant variability in the ASD group is consistent across all ERP component models.

### FSE and IE

We provide results for the FSE and IE analyses in Section “SM 3.3 FSE and IE Results” (Supplementary Tables 6–9) as the raw values showed poor test-retest stability over the 6 week period.

### Use for Stratification

For Aim 3, we possible that these markers, specifically the *a*N170L and *a*P100L to upright faces, may be useful in identifying a more homogeneous subgroup within children with ASD. We suggest a cutoff point based on the upper 10% from the TD group based on all available age-adjusted residual values (*a*N170L; Table 2, TD %90 = 29.20); this resulted in a subgroup of 62 ASD participants (29%) at T1 with slowed peak values for their age. For comparison, the *a*N170L for the age-adjusted slowed group (when *a*N170L > 29.2) had a mean (raw) N170L of 247.21 ms (SD 21.38. range 216–308) in contrast to the standard group (*a*N170L ≤ 29.2), which had a mean N170L of 194.30 ms (SD = 21.92, range 125–235). Those children with ASD and a slowed *a*N170L compared with those with ASD and a standard *a*N170L were of a similar age (*F*_1,212_ = 1.07, *p* = 0.30) but had lower Full Scale IQ, Verbal IQ, and NonVerbal IQ (*F*_1,212_ > 7.21, *p*s <0.01), and Face Memory SS (*F*_1,212_ = 4.97, *p* = 0.03). There were no differences between the ASD groups on measures of autism traits (ADOS CSS *F*_1,212_ = 3.04, *p* = 0.08; SRS SCI *F*_1,209_ = 0.36, *p* = 0.53; SRS RIRB *F*_1,210_ = 0.45, *p* = 0.50; PDDBI SocApp *F*_1,204_ = 0.40, *p* = 0.53) or adaptive skills (Vineland Socialization *F*_1,211_ = 0.030, *p* = 0.86, Communication *F*_1,211_ = 0.75, *p* = 0.39).

Using a similar strategy, for the P100L, a subgroup of 10% of TD participants with the slowest *a*P100L values (Table 2, TD %90 = 12.57) identified a subgroup of 49 ASD participants (23%) at T1 with slowed response for their age. For comparison, the raw P100L for the age-adjusted slowed group (when *a*P100L > 12.56) has a mean P100L response of 145.57 ms (SD 14.06 range 126–190); in contrast to the standard group (when *a*P100L ≤ 12.56) who had a mean P100L response of 114.49 ms (SD 9.92 range 90–135). Those children with ASD and a slowed *a*P100L compared with those with ASD and a standard *a*P100L were of a similar age (*F*_1,212_ = 0.312, *p* = 0.58); the stratification did not result in ASD subsamples differing on any of the behavioral measures included in this report such as the cognitive characterization measures (*F*_1,212_ ≤ 0.59, *p*s ≥ 0.51), measures of autism diagnosis or autistic behaviors (*F* ≤ 1.59, *p*s ≥ 0.21), or adaptive skills (*F*s <0.68, *p*s > 0.41).

## Discussion

### Upright Face Response

The primary aim of this analysis was to characterize the N170 latency and P100 latency age-related response to *upright faces* in children with ASD in relation to typical chronological age-related changes (Aim 1). Overall, and consistent with previous reports, N170 latencies to upright faces were slower in younger children and in children with ASD. Specifically, N170 latency in response to upright faces was associated with age, and decreased in peak latency of 6.57 ms per year across our 6–11-year-old sample. We also found that the P100L to upright faces was associated with age, with a 2.04 ms decrease in latency per year. This data suggests that the effects of age must be considered in evaluating “slowed” N170L and P100L in response to upright faces, as even within a relatively narrow age, the processes underlying these components are improving (in terms of latency) at different rates in childhood. Slight differences in age distribution could influence the identification of group differences. Important for age-based adjustments, the linear relationship between age and upright face-related ERP components make it relatively straightforward to develop age-adjusted values.

### Face Specificity Effect and Face Inversion Effects

We relegated the results for the FSE and the IE to Supplemental Materials due to the poor test-retest stability performance of these markers [e.g., ICC ≤ 0.5, (38)]. This lack of stability makes these difference score markers less useful for clinical trials (in this sample). It is possible that the markers might show greater stability under a different protocol (e.g., shorter time interval). However, given these results and in comparison to the relatively higher stability values of single-condition ERP markers, we do not suggest incorporation of these into clinical trials without further evaluation.

In regard to face processing more broadly, it has been suggested that the early neural selectivity or preference for faces as a category of stimuli is present by 4–5 years, and while the responses to the FaceUp and FaceInv mature and shorten in latency with age (14, 39), the differential processing of upright faces compared to objects or inverted faces, at either the P100 or the N170, did not seem to undergo age based differential change in our age group. In our analyses, we utilized difference scores for both the FSE and the IE, with a negative response reflected a faster FaceUp response in comparison to the contrast stimuli (HouseUp and FaceInv, respectively). Unexpectedly, across our sample, only the responses for P100L demonstrated a “face upright” preference, with a negative difference score in this pattern with ≥90% of TD participants (P100L IE 90.1%; FSE 100%) and ≥79% of ASD participants (P100L IE 79.8%; FSE 83.8%) demonstrating faster latencies to FaceUp than the contrast stimuli (Supplementary Table 6).

In contrast, the N170L did not show consistent differentiation of FaceUp and the contrast stimuli. N170L IE and FSE were, on average positive, with only a minority of the sample having values in the negative range (IE <0: TD 38%; ASD 36.8% FSE <0: TD 42%; ASD 45%). This confirms prior reports of an inconsistent IE in this age range. Further, there was not a while a clear trend in the association between age and the primary ERP components to upright faces, as the associations between age and the FSE and IE were minimal (N170L FSE) or negligible (P100L FSE, N170L IE, P100L IE). It has been suggested that the early neural selectivity or preference for faces as a category of stimuli is present by 4–5 years, and while the responses to the FaceUp and FaceInv mature and shorten in latency with age (14, 39), the differential processing of upright faces compared to objects or inverted faces, at either the P100 or the N170, did not seem to undergo age based differential change based on our analyses.

### Face Processing in ASD and Stratification

There are several potential uses for EEG biomarkers including stratification for inclusion, diagnosis or likelihood of developing ASD, prognostic, predictive, and surrogate endpoints (40). Both the N170 and P100 latency FaceUp biomarkers showed group discrimination, that is, with more positive or larger age-adjusted residual values in the ASD group compared to the than in the TD group; this response of slowed responses when adjusted for age, is similar to prior reports of slowed raw values [e.g., (23)]. However, there was significant overlap in the sample distributions, and it is unlikely that these ERP latency biomarkers would be useful for diagnostic purposes. As an alternative, we demonstrate that the using the age-based adjusted N170L, we can identify a group of children who have lower scores in face memory, cognitive, and verbal ability.

### Caveats

Of importance, if used as an inclusion variable, the biomarker would also need to consistently measure a trait of an individual. That is, if slowed face processing was a trait characteristic of a specific subgroup of autistic children, we would expect that this subgroup would be consistent across measurements. Our study was limited to having re-test values at +6 weeks, with stability influenced by trait, state, as well as measurement error. Our ICC values [N170L FaceUp ASD_T1−*T*2_ = 0.662; P100L FaceUp ASD_T1−*T*2_ = 0.680; (24)] suggest moderate stability over 6 weeks. Participants were not required to maintain stable treatments across the study and thus six-weeks may reflect a period of potential clinical change for some participants. Further evaluation of the usefulness of these ERP components as stratification variables at inclusion would benefit from assessment of reliability from periods more reflective of the time course of a clinical trial screening to baseline assessment period (e.g., ≤ 1 week).

This dataset, like many others in ASD research and clinical practice, included uneven distribution of sexes and the potential non-randomness of missing EEG data. Identifying whether or not age based adjustments also need to be sex or gender adjusted (as used in the SRS), will require inclusion of a larger sample of TD females. Further, while a sample of 60 autistic females is still higher than many other reports, there are substantial differences in the presentation of ASD between males and females and thus greater investigation of variability in ASD females is required.

In addition, missing data may not have been random. While we included 908–960 valid EEG data points (76–80% valid depending on the component and stimulus) making this one of the larger EEG datasets, there is more data loss than found in some other behavioral and experimental measures (e.g., eye tracking). For EEG, possible reasons for missing data at a given time point include ones that are common across measures (drop out), but also reflect the ways in which the child interacts with the experiment (boredom, behavioral non-compliance), and factors that may represent altered neural functioning (trial variability resulting in failure to show a peak response). As seen in Table 1, participants with no valid EEG data tended to have more impairing ASD symptoms, lower cognitive ability, and lower language ability. If symptom severity drives missingness of EEG data, EEG variables may have utility as a biomarker only within a certain range of symptom presentation.

Lastly, the biomarkers and behavioral/clinical assessments we include in this manuscript reflect a small selection of the potential variables that are available from the ABC-CT. Inclusion of alternative topographical responses (e.g., left hemisphere regions), amplitude of the ERP components or power of fundamental neural frequencies (e.g., alpha, theta), and measures of connectivity also need to be investigated in regard to their age based development. Further, exploration of these in relation to (novel) behavioral composites that may be more proximal to face attention will also be important. As the intent is to strengthen the toolbox of measures that can inform the clinical trial protocols, this manuscript represents a first step to identify if and how age based adjustments may be approached.

## Conclusion

Using the large dataset from the ABC-CT, we identify age related development in the latency of the P100 and N170 to upright faces and suggest that age-based adjustments will be necessary for implementation of these biomarkers in clinical trials. Further, we identified a clinically more impacted subgroup when using age-adjusted N170L values, suggesting the importance of considering peak latency values relative to chronological age-expected values, rather than absolute cutpoints.

## Data Availability Statement

The datasets presented in this study can be found in online repositories. The names of the repository/repositories and accession number(s) can be found at: Repository Data are available from NIMH NDA (#2288) (https://nda.nih.gov/edit_collection.html?id=2288).

## Ethics Statement

The studies involving human participants were reviewed and approved by Yale Human Research Protection Program. Written informed consent to participate in this study was provided by the participants' legal guardian/next of kin.

## Author Contributions

SW, IE, CS, DS, AN, SF, AL, FS, and JM contributed to the conceptualization, analysis and drafting the work, and editing of the manuscript. All authors made substantial contributions to the conception or design of the work and read and provided review and approval for publication of the content. All authors contributed to the intellectual content and review and approval of the manuscript.

## Funding

Support was provided by the Autism Biomarkers Consortium for Clinical Trials (ABC-CT) NIMH U19 MH108206 (JM) and NIMH R01 MH122428 (DS & Telesca).

## Conflict of Interest

GD is on the Scientific Advisory Boards of Janssen Research and Development, Akili Interactive, Inc, LabCorp, Inc, Roche Pharmaceutical Company, and Tris Pharma, and is a consultant to Apple, Gerson Lehrman Group, and Guidepoint Global, Inc. JMconsults with Customer Value Partners, Bridgebio, Determined Health, and BlackThorn Therapeutics, has received research funding from Janssen Research and Development, serves on the Scientific Advisory Boards of Pastorus and Modern Clinics, and receives royalties from Guilford Press, Lambert, and Springer. FS consults for Roche Pharmaceutical Company, Janssen Research and Development, and BioStream LLC. SW consults for Janssen Research and Development. The remaining authors declare that the research was conducted in the absence of any commercial or financial relationships that could be construed as a potential conflict of interest.

## Publisher's Note

All claims expressed in this article are solely those of the authors and do not necessarily represent those of their affiliated organizations, or those of the publisher, the editors and the reviewers. Any product that may be evaluated in this article, or claim that may be made by its manufacturer, is not guaranteed or endorsed by the publisher.
