# Supplementary material for: Identifying Age Based Maturation in the ERP Response to Faces in Children With Autism: Implications for Developing Biomarkers for Use in Clinical Trials
Source: Front Psychiatry. 2022 May 9;13:841236. doi: 10.3389/fpsyt.2022.841236 (PMC9126041; doi:10.3389/fpsyt.2022.841236)
Supplement: Supplementary file 1 [file Table_1.docx]

**Identifying age based maturation in the ERP response to faces in children with autism:**

**Implications for developing biomarkers for use in clinical trials**

**Supplemental Materials (SM)**

Section numbers reference the main manuscript sections. That is, main manuscript method section “2.0” corresponds to supplemental materials methods section “SM 2.0”.

**SM 2.0 Methods**

**SM 2.1 Protocol**

In the 2-day protocol, day 2 occurred from 1 to 14 days following day 1 (at each timepoint). Time 2 followed the T1-Day1 visit by 6 weeks (+/- 2 weeks). The actual temporal distance from the EEG visit T1-Day2 to T2-Day2 did not differ by group. Time 3 followed the T1-Day1 visit by 6 months (+/- 8 weeks). The actual temporal distance from T1-Day2 to T3-Day2 also did not differ by group.

**SM-Table-1. Mean, Standard Deviation (in parenthesis), and range of days between timepoints by group.**

|  | Total | ASD | TD | Main effect of group using unadjusted ANOVA |
| --- | --- | --- | --- | --- |
| T1-EEG to T2-EEG | 41.69 days (10.46)  17 to 90 | 41.61 (10.99)  17 to 90 | 41.89 (9.08)  21 to 64 | *F*_1,379_=.06, *p*=.81 |
| T1-EEG to T3-EEG | 166.23 days (11.97)  116 to 221 | 166.05 (12.35)  116 to 221 | 166.65 (11.11)  147 to 195 | F_1,364_=.20, *p*=.66 |

**SM 2.2 EEG Acquisition**

**SM 2.4 EEG Processing**

**SM 2.4.1 EEG Processing Data Loss**

Reasons for data loss are provided in SM-Table-2 and total data points available for each component are provided in SM-Table-3. The process steps below resulted in 94% inclusion rate for children in the TD group and 74% of children in the ASD group. Information on the valid number of artifact free, attended trials is presented in SM-Table-4.

**SM-Table-2: Data loss reason for the N170 Latency.**

Number of participants for Time 1 or all timepoints for the TD and ASD group who did not provide data and reason for data loss.

|  | Time 1  TD | Time 1 ASD | All  TD | All  ASD |
| --- | --- | --- | --- | --- |
| Enrolled | 119 | 280 | 357 | 840 |
| No EEG | 2 | 9 | 15 | 53 |
| Acquisition Invalid | 0 | 15 | 4 | 39 |
| Signal Invalid | 1 (<1%) | 42 (15%) | 2 (<1%) | 124 (14.8%) |
| <21 trials | 0 | 39 | 1 | 114 |
| Invalid Morphology | 1 | 3 | 1 | 10 |
| Valid | 116 | 214 | 336 | 624 |

**SM-Table-3: Data loss by ERP component.**

Number of participants with 1, 2, or 3 valid timepoints for the given ERP component. There were 399 participants in total (119 in the TD group, 280 in the ASD group). Face specificity effect (FSE) = latency in response to upright faces minus latency in response to upright houses. Inversion effect (IE) = latency in response to upright faces minus latency in response to inverted faces.

| ERP Component | Total datapoints | 1 Valid Timepoint  (ASD, TD) | 2 Valid Timepoints (ASD, TD) | 3 Valid Timepoints (ASD, TD) | No Valid Timepoints (ASD, TD) |
| --- | --- | --- | --- | --- | --- |
| N170L FaceUp | 960 (624,336) | 36 (31, 5) | 78 (70, 8) | 256 (151, 105) | 29 (28, 1) |
| P100L FaceUp | 959 (623,336) | 35 (30, 5) | 78 (70, 8) | 256 (151, 105) | 30 (29, 1) |
| N170 FSE | 908 (584,324) | 46 (38, 8) | 86 (75, 11) | 230 (132, 98) | 37 (35, 2) |
| P100 FSE | 908 (585,323) | 44 (36, 8) | 87 (75, 12) | 230 (133, 97) | 38 (36, 2) |
| N170 IE | 946 (612,334) | 37 (31, 6) | 81 (73, 8) | 249 (145, 104) | 32 (31, 1) |
| P100 IE | 951 (619,332) | 37 (30, 7) | 76 (68, 8) | 254 (151, 103) | 32 (31, 1) |

**SM 2.4.2 EEG Processing Peak Identification**

There is inconsistency in the identification of the N170 peak in prior reports. For example, Taylor and colleagues analyzed the N170b or second peak as it was present more consistently across the ages included in their report (Itier & Taylor, 2004; Taylor et al., 2004). In contrast, Keufner et al. analyzed the first negative peak after the P100 (Kuefner et al., 2010). Mares et al. did not abstract the peak of the N170 due to peak shape variability (Mares et al., 2020). In summary, there is currently no clear standard for quantification of the N170, particularly related to the variability in waveform morphology that reflects bifid peaks. The extent to which a non-monotonic P100-N170 morphology might be related to development or atypical processing is unknown.

Manuals for deriving the P100 and N170 from the assay and the peak picking protocol are available on request from the author. In summary, an automated algorithm was first deployed. Peak detection calculations were run on the specific region of interest. In the first step, the algorithm identified the P100 as the most positive peak within a pre-specified window. The N170 is then defined as the largest negative peak following the P100 within the latency window of 120 to 400 msec. Starting from the P100 peak, the “peak finder” moves in the positive time direction to find the next most negative point at which slope = 0. The median latency of the peak is identified using 20 different smoothing parameters. After identification of the median peak latency from the “smoothed” data loops, the program examines the unsmoothed data starting from the median value, going in both positive and negative time directions. Of the peak returned from this bi-directional search, the peak (where slope = 0) closer to the median value is chosen as the N170.

Visual inspection of all waveforms (by individual, by timepoint, by region of interest, by condition) was included as the automatic peak identification program could misidentify the component(s) in cases wherein large oscillations obscured the components, when the morphology was represented by a wide or shallow peak, and/or when a double peak was present. For peak review, a standard training protocol (developed by SJW and AN) was employed for each staff member, including review of the manual that included the peak definitions and ~30 coded samples, as well as group discussion of 20 example waveforms from our confirmation study. Each team member then consensus coded ~50 cases with SJW. After achieving agreement on the cases, the staff member was provided with the remaining data set. All peaks were double coded without reference to group, age site, or other demographic characteristics. Reliability was checked by JB and discrepancies were moderated by SJW.

This visual peak verification was done by visual inspection using a graphical user interface in Matlab. Each waveform was plotted in a window of +/- 20㎶ (or re-plotted within a window of +/- 40 ㎶ for valid-appearing waveforms that exceeded the plotting window).  For the ABC-CT, peak values were marked valid if they were within the latency window (P100 :  60 to 200; N170 : 120 to 400); a point of no slope [slope = 0]; the peak was the largest peak within the temporal window; the peak has a resolution of a specific amplitude size, before and after the peak. That is, within the peak window, wherein the peak is slope = 0, the amplitude change is ≥2㎶ before and after the P1 and N170 peaks. The peak or morphology was determined to be invalid if (a) the peak was outside of the predefined window of interest; (b) the baseline activity from -100 to 0 msec exceeded a 20µV range; (c) if 3 cycles of repetitive oscillatory activity was present (between –200msec to 600msec) and exceeded the amplitude of the P100 or N170 making the P100 or N170 peaks unclear within the larger (e.g., alpha) oscillations; and (d) the slope preceding and following the peak was ≤2 µV within the predefined window.

**SM-Table-4: Average number of artifact free attended trials for the face upright response for all included data points.**

A minimum of 21 trials for face upright was required for inclusion in the analyses.

|  | Mean  M (SD) | Median | Min | Max | 25% | 75% |
| --- | --- | --- | --- | --- | --- | --- |
| TD | 53.5 (11.37) | 55 | 21 | 71 | 46 | 63 |
| ASD | 47.3 (13.41) | 49 | 21 | 71 | 36 | 58 |

**SM 2.5 Analytic Plan**

**SM 2.5.1 Age Based Development**

**SM-Table-5: AIC values for mixed effect models of age-based changes in ERP components.**

Model 1 was a random intercept model with fixed effects of timepoint, group, and mean-centered age at testing, and subject level random intercepts with different random effect variance structures for TD and ASD. Model 2 was a random intercept model with fixed effects of timepoint, group, mean-centered age at testing, and interaction between group and mean-centered age at testing. Model 2 included subject level random intercepts with different random effect variance structures for TD and ASD. Model 3 was a random intercept model with fixed effects of timepoint, group, mean-centered age at testing, and quadratic mean-centered age at testing. Model 3 included subject level random intercepts with different random effect variance structures for TD and ASD

| Model | N170L FU | P100L FU |
| --- | --- | --- |
| Model 1 | 8951 | 7624 |
| Model 2; Interaction | 8961 | 7636 |
| Model 3; Quadratic | 8975 | 7649 |

**SM 3.3 FSE and IE Results**

To provide context to the analyses related to FSE and IE and to match to our original report, we include Time 1 ANCOVAs with child age, number of trials (for FaceUp), sex (male/female) and full scale IQ as covariates (SM-Table-5). For the FSE at Time 1, data was available on 207 children with ASD and 113 children with TD. Neither the N170L FSE nor the P100L FSE responses differed by group. However, using one-sample t-tests, compared to a test value of 0, the N170L was faster to houses than faces (positive value) for the TD group and at a trend level for the ASD group. The P100L was faster to upright face than houses in both groups (represented by a negative FSE value, *p*s<.001)(SM-Table-5).

The IE data included 212 children with ASD and 115 children with TD at Time 1. The N170L IE significantly differed between the two groups. The ASD group showed a faster response to houses than faces (positive value, *p*<.0001), while the TD group did not show a difference between the two groups (*p*=.105). In contrast, the P100L IE did not differ between the groups but both groups showed a faster response to upright faces compared to inverted faces (negative value, *p*s<.001).

We then assessed short-term stability of FSE and IE biomarkers in both groups from T1 to T2 using intraclass correlation (ICC) via two-way mixed effect models with absolute agreement (SM-Table-6). A priori acceptability criteria for six-week stability was defined as ICCs ≥.5 for moderate and ≥.75 as high values (Ciccehtti, 1994; Donner & Koval, 1980). The P100L FSE was the only variable that showed moderate stability; although the difference in stability for younger vs older ASD is concerning. As well, all other difference score biomarkers (N170L FSE, N170L IE, and P100L IE) did not show acceptable stability for use in a clinical trial.

**SM-Table-6: Means and standard deviations (in parentheses) and group comparisons for Time 1 FSE and IE biomarkers.**

| Time 1 | ASD  M (SD) | ASD One-Sample T-Test (test value=0) | TD  M (SD) | TD One-Sample T-Test (test value=0) | Group Differences  ANCOVA |
| --- | --- | --- | --- | --- | --- |
| N170L FSE | M=4.13 (33.62) | t=1.768, *p*=.079 | M=5.61 (22.59) | *t*=2.640, *p*=.009 | *F*_1,314_=.499, *p*=.480, *ηp*^2^=.002 |
| P100L FSE | M=-10.49 (15.28) | *t*=-9.877, *p*<.001 | M=-12.81 (9.42) | *t*=-14.447, *p*<.001 | *F*_1,314_=2.13, *p*=.146, *ηp*^2^=.007 |
| N170L IE | M=12.81 (31.71) | *t*=5.881, *p*<.001 | M=4.39 (28.79) | *t*=1.636, *p*=.105 | *F*_1,321_=5.49, *p*=.020, *ηp*^2^=.017 |
| P100 IE | M=-4.64 (13.34) | *t*=-.5081, *p*<.001 | M=-6.28 (2.07) | *t*=-9.483, *p*<.001 | *F*_1,321_=1.06, *p*=.304, *ηp*^2^=.003 |

**SM-Table-7. Six-week stability (ICC) from Time 1 to Time 2 for the FSE and IE biomarkers.**

Values provided are for the TD and ASD included sample and then for ASD subgroups by age. We do not provide subgroups by IQ as the lower IQ (≤75) group was too small (n≤10).

| Time 1 to Time 2  ICC | TD | ASD | ASD <8.5y | ASD ≥8.5y |
| --- | --- | --- | --- | --- |
| N170L FSE | .405 | .337 | .374 | .207 |
| P100L FSE | .592 | .567 | .714 | .391 |
| N170L IE | .484 | .495 | .284 | .607 |
| P100L IE | .328 | .284 | .221 | .425 |

**SM 3.1 Age based development using Linear Mixed Models (LMM)**

**3.1.1 Face Specificity Effect**

The FSE reflects the comparison between the speed of processing of FaceUp and HouseUp. The N170 FSE latency was an estimated -.011 msec (95% CI: -0.015, -0.0071; SM-Table-8, left), such that the difference between FaceUp and HouseUp was becoming more negative with age, likely reflecting the faster latency of the N170L FaceUp (noted above) and less or no change in the N170L HouseUp response. Between-participant variance for N170L FSE (*SD*_ASD_=18.9, *SD*_TD_=12.6) was greater in the ASD group than in the TD group.

The age effect for P100L FSE was an estimated .00054 msec (95% CI: -0.0023, 0.0012), which was non-significant (SM-Table-8, right). Between-participant variance for P100L FSE was greater in the ASD group (*SD*_ASD_=8.4) than in the TD group (*SD*_TD_=4.4).

**SM-Table-8: Random and fixed effects for N170 latency and P100 latency to Face Specificity Effect.**

Mixed effect model fits a random y-intercept with differing variances for typically developing (TD) and autism spectrum disorder (ASD) groups.

| N170 Latency FSE | | | P100 Latency FSE | | |
| --- | --- | --- | --- | --- | --- |
| 908 observations, 362 participants | | | 908 observations, 361 participants | | |
| Fixed  Effects | Estimate (95% CI) | *t*  value | Fixed  Effects | Estimate (95% CI) | *t*  value |
| Intercept | 3.5 (-0.95, 7.85) | 1.5 | Intercept | -13.51 (-15.31, -11.70) | -14.7 |
| Age | -0.011 (-0.015,-0.0071) | -5.3 | Age | -0.00054 (-0.0023, 0.0012) | -0.6 |
| ASD group | 1.71 (-3.20, 6.64) | 0.68 | ASD group | 3.38 (1.32, 5.43) | 3.2 |
| T2 | -2.92 (-7.13, 1.29) | -1.4 | T2 | 1.25 (-0.57, 3.07) | 1.3 |
| T3 | -0.86 (-5.24, 3.53) | -0.39 | T3 | 2.34 (0.46, 4.23) | 2.4 |
| Random Effects | Variance (SD) |  | Random Effects | Variance (SD) |  |
| TD | 158.6 (12.6) |  | TD | 19.2 (4.4) |  |
| ASD | 358.7 (18.9) |  | ASD | 71.1 (8.4) |  |
| Residual | 701.1 (26.5) |  | Residual | 130.4 (11.4) |  |

**3.1.2 Inversion effect**

The IE reflects the comparison between the speed of processing of FaceUp and FaceInv. The age effect for the N170 IE Latency was .0018 msec (95% CI: -0.024, 0.0059) and reflects a nonsignificant age effect (SM-Table-9, left). Between-participant variance for the N170L IE was greater in the ASD group (*SD*_ASD_=20.8) than in the TD group (*SD*_TD_=10.4).

The average P100 IE Latency was an estimated 0.0035 msec (95% CI: 0.0020, 0.0050; SM-Table-9, right), such that a more positive value reflects a P100L FaceInv response that was becoming faster at a greater rate than the P100L FaceUp response (noted above). The P100L IE between-participant variance was also greater in the ASD group than in the TD group but due to the extremely small between-participant variance in the TD group (approaching 0), a full model with group-specific y-intercept variance did not yield stable results and was redacted to a simpler model with combined y-intercept variance.

**SM-Table-9: Random and fixed effects for Inversion Effect.** Age at EEG was a fixed effect in all models and participant ID was a random effect in all models.

| N170 Latency IE | | | P100 Latency IE | | |
| --- | --- | --- | --- | --- | --- |
| 946 observations, 367 participants | | | 951 observations, 367 participants | | |
| Fixed  Effects | Estimate (95% CI) | *t* value | Fixed  Effects | Estimate (95% CI) | *t* value |
| Intercept | 6.96 (2.62, 11.3) | 3.1 | Intercept | -6.55 (-8.28, -4.82) | -7.4 |
| Age at EEG | 0.0018 (-0.0024, 0.0059) | 0.82 | Age at EEG | 0.0035 (0.0020, 0.0050) | 4.7 |
| ASD group | 4.94 (-0.0077, 9.89) | 2.0 | ASD group | 2.11 (0.29, 3.92) | 2.3 |
| T2 | -3.03 (-7.41, 1.35) | -1.4 | T2 | 0.65 (-0.94, 2.25) | 0.80 |
| T3 | -1.68 (-6.19, 2.83) | -0.73 | T3 | 1.03 (-0.61, 2.67) | 1.2 |
| Random Effects | Variance (SD) |  | Random Effects | Variance (SD) |  |
| TD | 107.4 (10.4) |  | Participant* | 28.9 (5.4) |  |
| ASD | 433.7 (20.8) |  |  |  |  |
| Residual | 782.0 (28.0) |  | Residual | 105.4 (10.3) |  |

* Data complexity does not support differing variance structures for ASD and TD groups.

**SM 3.2 Age Adjusted Residuals**

**SM-Table-10: Raw Mean, Standard Deviation, and range of the N170L and P100L for response to FaceUp, FSE, and IE.** The model used to calculate residuals was a mixed effect model fitted in the TD group. Age was a fixed effect and participant ID was a random effect. Skewness is close to 0 when the distribution is symmetrical, negative when the left tail of the distribution is longer, and positive when the right tail of the distribution is longer. Larger values of kurtosis indicate heavier tails (kurtosis=3 for a univariate normal distribution). Note, the FaceUp responses duplicate Table-2 and are provided for context.

|  | **FaceUp**  **N170L** | |  | **FaceUp**  **P100L** | |  | **FSE**  **N170L** | | **FSE**  **P100L** | | **IE**  **N170L** | | **IE Raw**  **P100L** | |
| --- | --- | --- | --- | --- | --- | --- | --- | --- | --- | --- | --- | --- | --- | --- |
|  | **TD** | **ASD** | | **TD** | **ASD** | | **TD** | **ASD** | **TD** | **ASD** | **TD** | **ASD** | **TD** | **ASD** |
| **N** | 336 | 624 | | 336 | 623 | | 324 | 584 | 323 | 585 | 334 | 612 | 332 | 619 |
| **Missing** | 21 | 216 | | 21 | 217 | | 33 | 256 | 34 | 255 | 23 | 228 | 25 | 221 |
| **Mean** | 193.60 | 206.23 | | 117.57 | 121.74 | | 2.42 | 3.39 | -12.29 | -8.98 | 5.43 | 10.26 | -5.97 | -3.62 |
| **Median** | 193 | 203.5 | | 117 | 119 | | 3.5 | 3.5 | -11 | -9 | 4 | 7 | -6 | -5 |
| **SD** | 27.13 | 34.17 | | 13.12 | 16.89 | | 26.67 | 34.47 | 10.95 | 14.78 | 25.41 | 36.43 | 7.47 | 13.55 |
| **Skewness** | 0.33 | 0.92 | | 0.96 | 1.20 | | -1.81 | -0.13 | -0.76 | 0.10 | -0.75 | 0.27 | 0.11 | 1.27 |
| **Std. Error of Skewness** | 0.13 | 0.010 | | 0.13 | 0.10 | | 0.14 | 0.10 | 0.14 | 0.10 | 0.13 | 0.10 | 0.13 | 0.10 |
| **Kurtosis** | 0.26 | 2.97 | | 4.35 | 2.07 | | 10.73 | 7.44 | 6.08 | 6.59 | 6.43 | 5.09 | 14.13 | 11.69 |
| **Std. Error of Kurtosis** | 0.27 | 0.20 | | 0.27 | 0.20 | | 0.27 | 0.20 | 0.27 | 0.20 | 0.27 | 0.20 | 0.27 | 0.20 |
| **Minimum** | 125.0 | 125.0 | | 82.0 | 82.0 | | -186.0 | -177.0 | -81.0 | -87.0 | -140.0 | -170.0 | -56.0 | -80.0 |
| **Maximum** | 276.0 | 393.0 | | 189.0 | 192.0 | | 71.0 | 198.0 | 33.0 | 73.0 | 86.0 | 197.0 | 44.0 | 73.0 |
| **Percentiles 10** | 158.7 | 166.5 | | 103.7 | 104.0 | | -22.0 | -27.0 | -24.0 | -22.0 | -13.5 | -21.0 | -13.0 | -14.0 |
| **Percentiles 25** | 177.0 | 186.0 | | 110.0 | 111.0 | | -8.75 | -11.0 | -18.0 | -16.0 | -4.0 | -5.0 | -9.0 | -9.0 |
| **Percentiles 30** | 181.0 | 190.0 | | 112.0 | 113.0 | | -6.0 | -8.0 | -16.0 | -14.0 | -2.0 | -3.0 | -9.0 | -8.0 |
| **Percentiles 50** | 193.0 | 203.5 | | 117.0 | 119.0 | | 3.5 | 3.5 | -11.0 | -9.0 | 4.0 | 7.0 | -6.0 | -5.0 |
| **Percentiles 70** | 204.9 | 219.0 | | 122.0 | 126.0 | | 12.0 | 14.0 | -8.0 | -5.0 | 11.0 | 20.0 | -4.0 | -2.0 |
| **Percentiles 75** | 208.0 | 224.0 | | 124.0 | 128.0 | | 14.0 | 19.0 | -6.0 | -3.0 | 15.0 | 24.0 | -3.0 | -1.0 |
| **Percentiles 90** | 229.30 | 246.0 | | 130.30 | 145.0 | | 32.0 | 40.0 | -2.0 | 5.0 | 32.5 | 52.70 | 0.70 | 5.0 |

**SM-Table-11: Mean, Standard Deviation, and range of the (residualized or age-adjusted) *a*N170L and *a*P100L for response to FaceUp, FSE, and IE.** The model used to calculate residuals was a mixed effect model fitted in the TD group. Age was a fixed effect and participant ID was a random effect. Skewness is close to 0 when the distribution is symmetrical, negative when the left tail of the distribution is longer, and positive when the right tail of the distribution is longer. Larger values of kurtosis indicate heavier tails (kurtosis=3 for a univariate normal distribution). Note, the FaceUp responses duplicate Table-2 and are provided for context.

|  | **FaceUp**  ***a*N170L** | |  | **FaceUp**  ***a*P100L** | |  | **FSE**  ***a*N170L** | | **FSE**  ***a*P100L** | | **IE**  ***a*N170L** | | **IE**  ***a*P100L** | |
| --- | --- | --- | --- | --- | --- | --- | --- | --- | --- | --- | --- | --- | --- | --- |
|  | **TD** | **ASD** | | **TD** | **ASD** | | **TD** | **ASD** | **TD** | **ASD** | **TD** | **ASD** | **TD** | **ASD** |
| **N** | 336 | 624 | | 336 | 623 | | 324 | 584 | 323 | 585 | 334 | 612 | 332 | 619 |
| **Missing** | 21 | 216 | | 21 | 217 | | 33 | 256 | 34 | 255 | 23 | 225 | 25 | 221 |
| **Mean** | 0.19 | 14.01 | | 0.17 | 4.76 | | 0.27 | 1.69 | 0.10 | 3.54 | 0.08 | 4.68 | 0.04 | 2.25 |
| **Median** | -1.82 | 11.45 | | -0.38 | 1.95 | | 1.55 | 0.97 | 0.80 | 3.48 | -1.09 | 1.30 | -0.10 | 0.96 |
| **SD** | 25.04 | 32.31 | | 12.60 | 16.76 | | 26.10 | 33.70 | 10.88 | 14.90 | 25.39 | 36.47 | 7.38 | 13.36 |
| **Skewness** | 0.47 | 1.14 | | 0.86 | 1.23 | | -1.90 | -0.10 | -0.77 | 0.15 | -0.73 | 0.25 | 0.23 | 1.27 |
| **Std. Error of Skewness** | 0.13 | 0.10 | | 0.13 | 0.10 | | 0.14 | 0.10 | 0.14 | 0.10 | 0.13 | 0.10 | 0.13 | 0.10 |
| **Kurtosis** | 0.93 | 3.54 | | 4.51 | 2.16 | | 11.58 | 7.82 | 5.95 | 6.52 | 6.29 | 5.09 | 15.10 | 11.82 |
| **Std. Error of Kurtosis** | 0.27 | 0.20 | | 0.27 | 0.20 | | 0.27 | 0.20 | 0.27 | 0.20 | 0.27 | 0.20 | 0.27 | 0.20 |
| **Minimum** | -68 | -60 | | -41 | -32 | | -187 | -184 | -68 | -73 | -146 | -178 | -50 | -74 |
| **Maximum** | 88 | 185 | | 69 | 72 | | 77 | 195 | 44 | 86 | 83 | 190 | 51 | 78 |
| **Percentiles 10** | -28.61 | -23.21 | | -13.27 | -12.61 | | -22.81 | -29.07 | -11.28 | -10.27 | -19.98 | -25.62 | -6.87 | -7.74 |
| **Percentiles 25** | -15.51 | -7.15 | | -6.85 | -5.24 | | -9.58 | -12.09 | -5.55 | -3.63 | -9.77 | -11.11 | -3.13 | -3.23 |
| **Percentiles 30** | -13.82 | -2.60 | | -5.53 | -3.83 | | -6.87 | -9.32 | -3.78 | -2.01 | -6.83 | -8.89 | -2.46 | -1.94 |
| **Percentiles 50** | -1.82 | 11.45 | | -0.38 | 1.95 | | 1.55 | 0.97 | 0.80 | 3.48 | -1.09 | 1.30 | -0.10 | 0.96 |
| **Percentiles 70** | 12.75 | 26.01 | | 5.18 | 8.77 | | 9.39 | 12.39 | 5.35 | 8.00 | 6.10 | 13.98 | 2.50 | 4.16 |
| **Percentiles 75** | 16.90 | 30.97 | | 6.77 | 10.41 | | 12.51 | 15.97 | 6.47 | 9.40 | 9.47 | 18.43 | 3.09 | 4.90 |
| **Percentiles 90** | 29.20 | 50.68 | | 12.57 | 27.28 | | 26.98 | 37.47 | 10.87 | 17.47 | 27.06 | 45.88 | 6.27 | 11.44 |

**Supplemental Materials (SM) Figures**

**SM-Figure-1. Schematic of trial structure for three trials.** Each trial consisted of a fixation crosshair (500-650ms), stimulus (500ms), and blank screen (500-650ms), resulting in trial lengths (randomly) varying between 1500 to 1800 msec. Facial images depicted are 01F and 18F via publishing permissions; publishing permissions are not available for 07F, 13F, 17F, which were used in the assay (Tottenham et al., 2002, 2009). House images were purchased from Shutterstock ([www.shutterstock.com](http://www.shutterstock.com); 252868810, 150435080, 58015144).

**
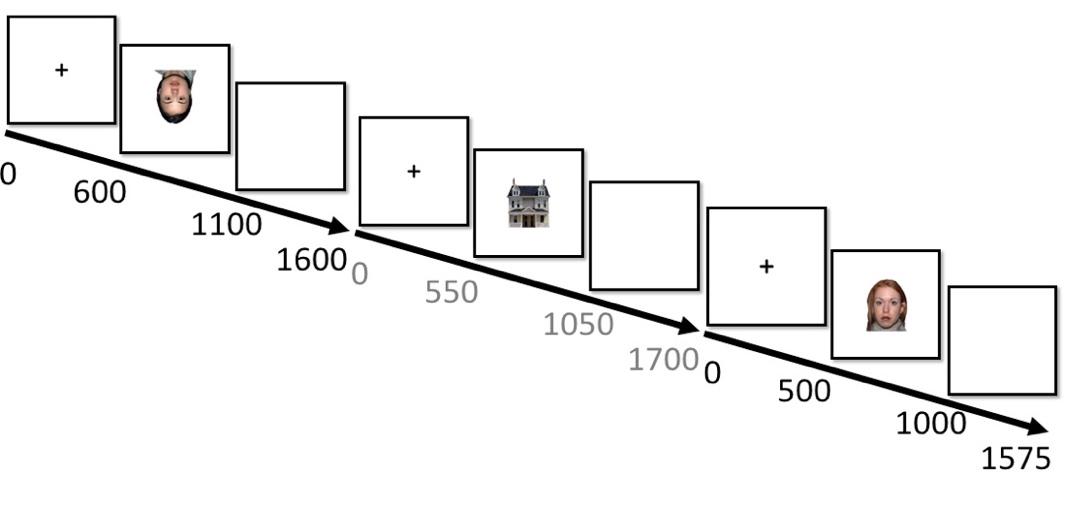
**

**SM-Figure 2: Layout for the HydroCel Geodesic Sensor Net.** Nets utilized in this study did not consistently have electrodes 125-128 (eye and face electrodes) and thus these are removed from the channel map. We focused on the right posterior-temporal region (RPT) depicted in grey, which was created by averaging 5 channels (89, 90, 91, 95, 96) for each segment.

**
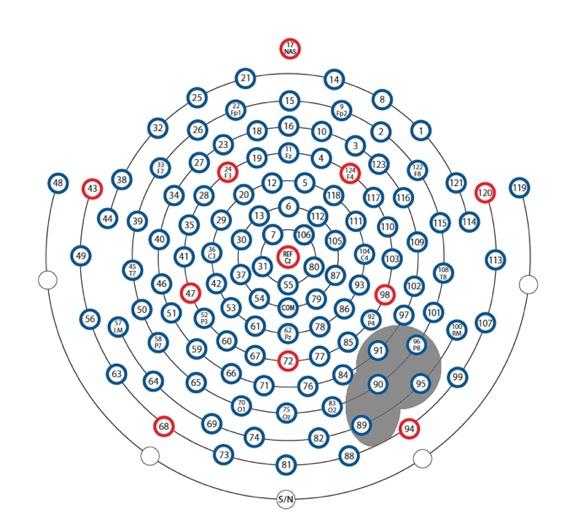
**

**SM-Figure-3: Grand Average Waveform by timepoint.** Response from the ASD (green) and TD (blue) group, for each timepoint for FaceUp (line) and HouseUp (dotted line).

**
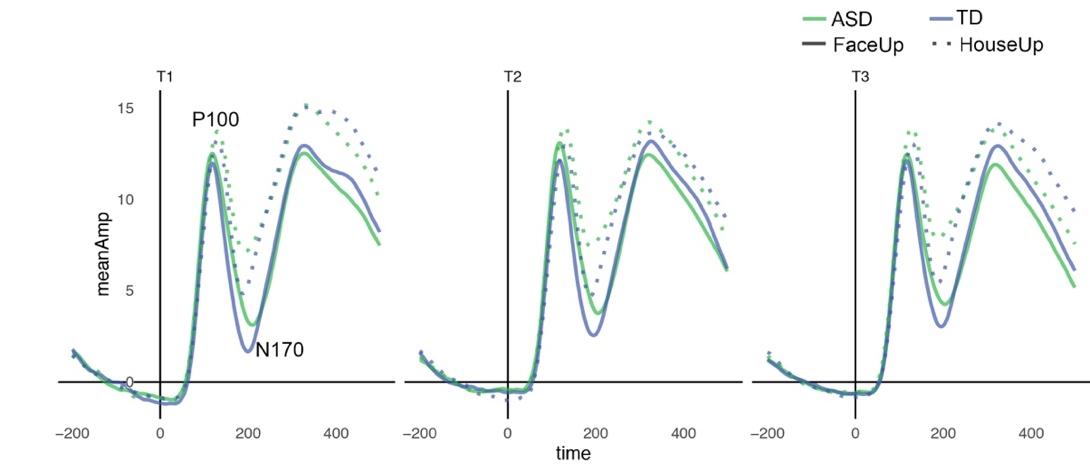
**

**SM-Figure 4: *a*N170L Face Specificity Effect.** Age-adjusted or residualized N170 Latency Face Specificity Effect. FSE refers to the difference between upright faces – upright houses; negative raw values reflect faster responses to upright faces with larger values reflecting greater differentiation. Age adjustment was calculated using a random intercept model with the fixed effect of age at testing and random effect of participant ID fitted to all available N170L specification values for TD participants. (A and D): The red line in column 1 indicates predicted values of N170L specification based on the fitted model, while the blue line indicates the locally estimated scatterplot smoothing (LOESS) for each group. (B, C, E, F): Columns 2 and 3 show residuals values calculated using the fitted model. Black lines in columns 2 and 3 indicate a cutoff point derived from the upper 10% of all age-adjusted N170L specification scores in the TD group. (A, B, D, E): In columns 1 and 2, data points greater than that cutoff point are colored in black.


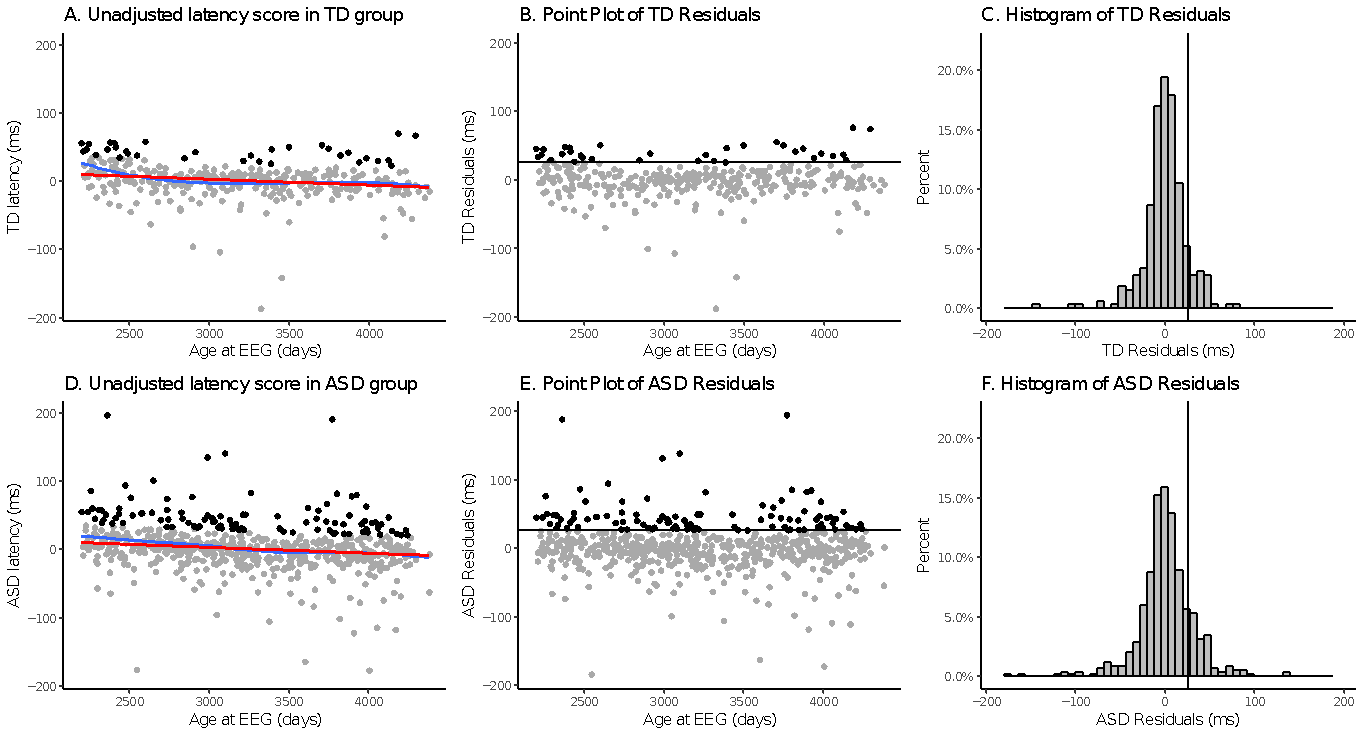


**SM-Figure-5: *a*P100L Face Specificity Effect.**Age-adjusted or residualized P100L FSE. FSE refers to the difference between upright faces – upright houses; negative raw values reflect faster responses to upright faces with larger values reflecting greater differentiation. Age adjustment was calculated using a random intercept model with the fixed effect of age at testing and random effect of participant ID fitted to all available P100L FSE values for TD participants. (A and D): The red line in column 1 indicates predicted values of P100L FSE based on the fitted model, while the blue line indicates the locally estimated scatterplot smoothing (LOESS) for each group. (B, C, E, F): Columns 2 and 3 show residuals values calculated using the fitted model. Black lines in columns 2 and 3 indicate a cutoff point derived from the upper 10% of all age-adjusted P100L FSE scores in the TD group. (A, B, D, E): In columns 1 and 2, data points greater than that cutoff point are colored black.


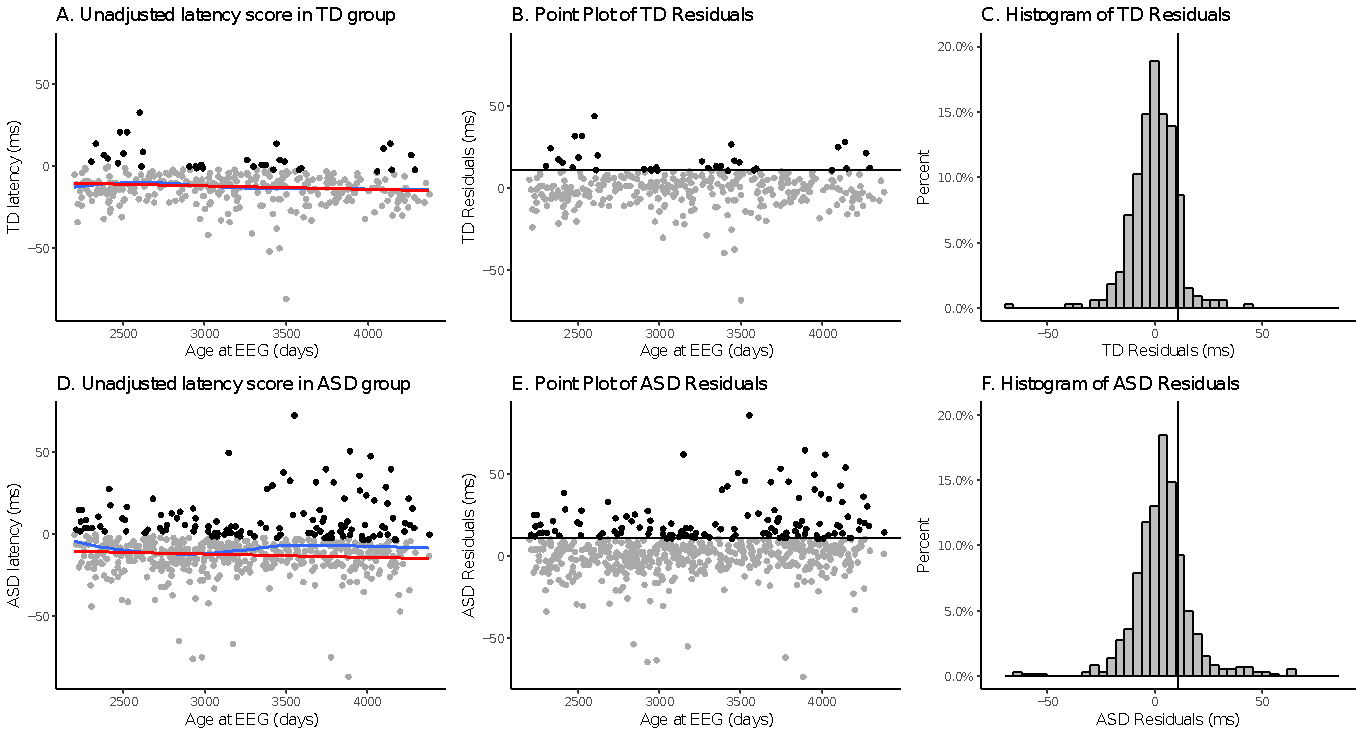


**SM-Figure 6: Residualized N170L Inversion Effect.**Age-adjusted N170L IE. The inversion effect refers to the difference between upright faces - inverted faces; negative raw values reflect faster responses to upright faces with larger values reflecting greater differentiation. Age adjustment was calculated using a random intercept model with the fixed effect of age at testing and random effect of participant ID fitted to all available N170L inversion values for TD participants. (A and D): The red line in column 1 indicates predicted values of N170L inversion based on the fitted model, while the blue line indicates the locally estimated scatterplot smoothing (LOESS) for each group. (B,C,E,F): Columns 2 and 3 show residuals values calculated using the fitted model. Black lines in columns 2 and 3 indicate a cutoff point derived from the upper 10% of all age-adjusted N170L inversion scores in the TD group. (A, B, D, E): In columns 1 and 2, data points greater than that cutoff point are colored black.


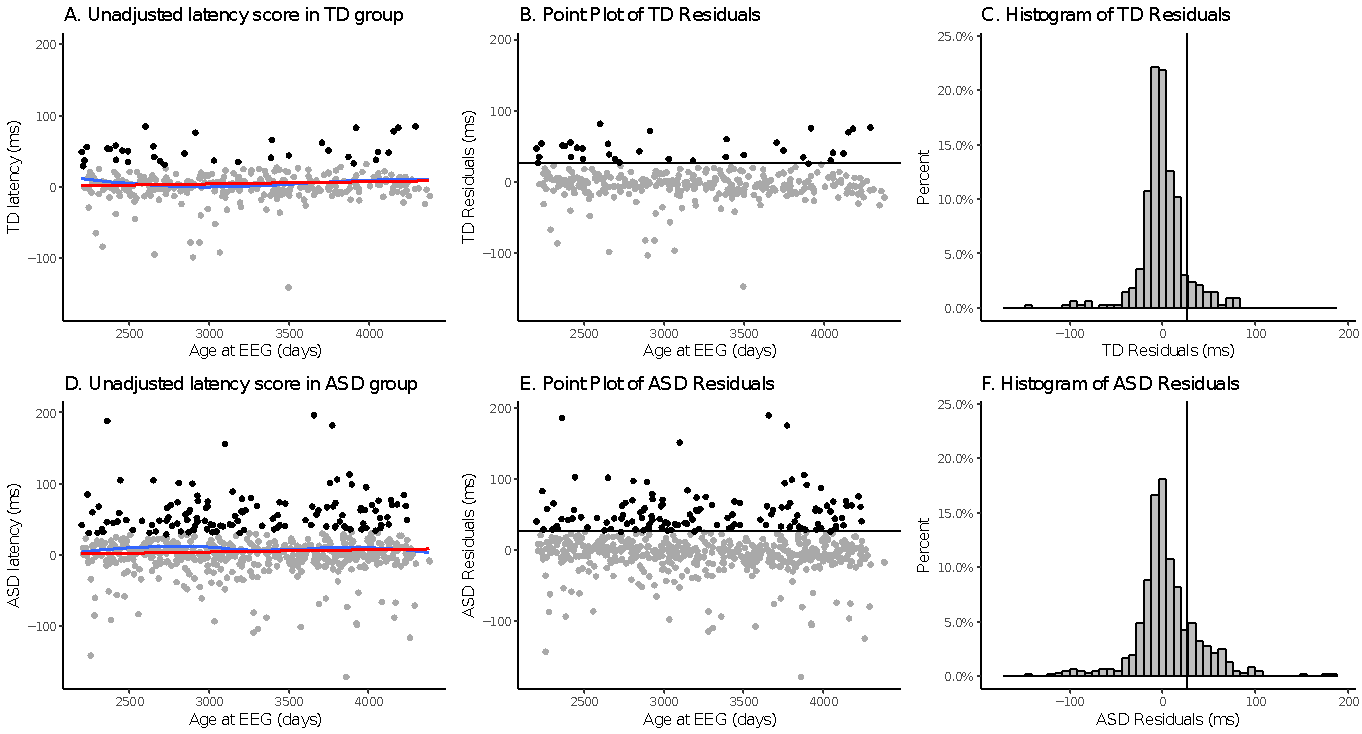


**SM-Figure-7: Residualized P100L Inversion Effect.**Age-adjusted P100 IE. The IE refers to the difference between upright faces - inverted faces; negative raw values reflect faster responses to upright faces with larger values reflecting greater differentiation. Age adjustment was calculated using a random intercept model with the fixed effect of age at testing and random effect of participant ID fitted to all available P100L inversion values for TD participants. (A and D): The red line in column 1 indicates predicted values of P100L inversion based on the fitted model, while the blue line indicates the locally estimated scatterplot smoothing (LOESS) for each group. (B, C, E, F): Columns 2 and 3 show residuals values calculated using the fitted model. Black lines in columns 2 and 3 indicate a cutoff point derived from the upper 10% of all age-adjusted P100L inversion scores in the TD group. (A, B, D, E): In columns 1 and 2, data points greater than that cutoff point are colored black.

**
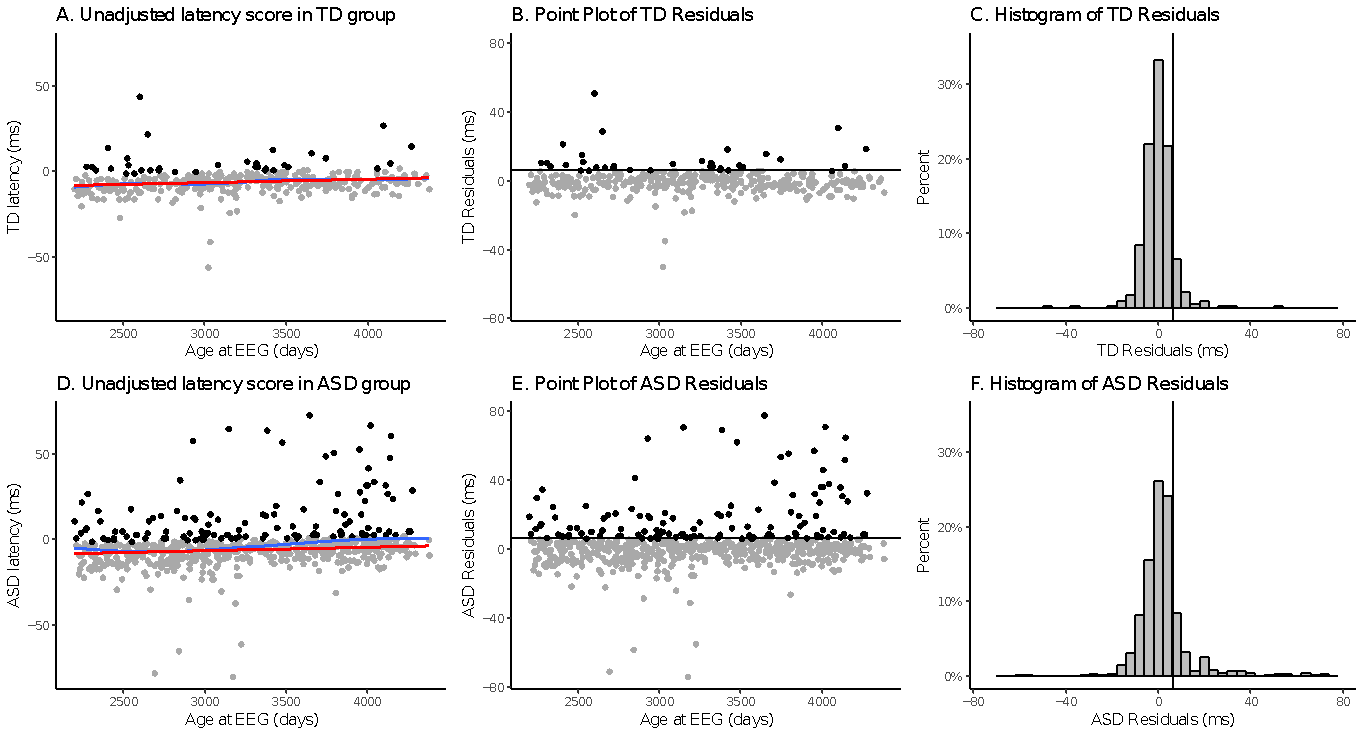
**

**References**

Cicchetti, D. V. (1994). Guidelines, criteria, and rules of thumb for evaluating normed and standardized assessment instruments in psychology. Psychol. Assess. 6, 284–290. doi: 10.1037/1040-3590.6.4.284

Donner A, Koval JJ. The estimation of intraclass correlation in the analysis of family data. *Biometrics*. 1980;36(1):19-25.
